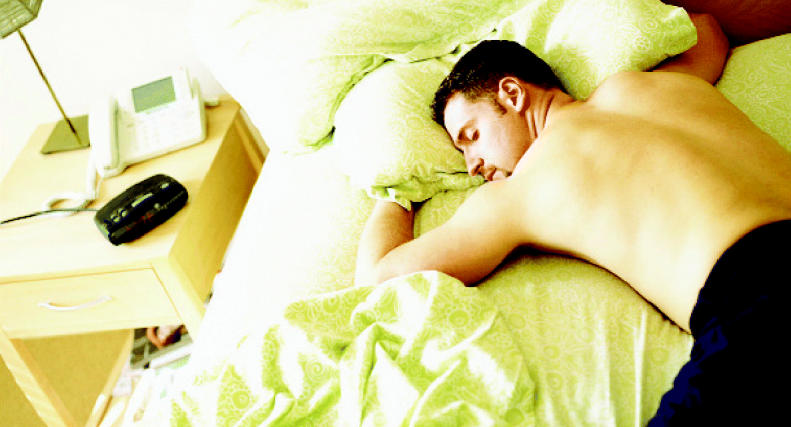# Headliners: Proteins: Timeless Protein Plays a Role in Coupling Cell Cycle and Circadian Rhythm

**Published:** 2005-09

**Authors:** Jerry Phelps

Ünsal-Kaçmaz K, Mullen TE, Kaufmann WK, Sancar A. 2005. Coupling of human circadian and cell cycles by the Timeless protein. Mol Cell Biol 25:3109–3116.

In mammals, the Timeless protein is necessary for proper functioning of circadian rhythm, the predictable “internal body clock” that regulates the 24-hour cycle of biological processes in animals and plants. The protein is also evolutionarily related to many cell cycle control proteins, which mediate cellular pathways that are activated by environmental changes or cellular injury, resulting in a protective response. In the current study, NIEHS grantee William K. Kaufmann and colleagues at the University of North Carolina–Chapel Hill set out to determine whether the Timeless protein may itself function as a core component of the human cell cycle.

Disruptions in circadian rhythm have been implicated in a variety of diseases and conditions from common jet lag to cancer. The cell cycle is the orderly sequence of events by which a cell duplicates its contents and divides into two. Both systems have pervasive effects on physiology at the levels of the cell, organ, and organism.

Although the two systems have distinct regulatory mechanisms, there is growing evidence that they are linked. Most mammalian cells function on an approximate 24-hour cell cycle, and the circadian clock has been implicated in regulating the phases of cell division. This linkage is important for a new field of research and medicine known as chronotherapy, which aims to coordinate the delivery of chemotherapeutic drugs with the circadian and cell cycles to maximize drug efficacy while minimizing side effects.

Kaufmann and colleagues found that the human Timeless protein interacted with both Cry2 (a confirmed circadian clock protein) and Chk1 kinase (a cell cycle checkpoint protein). Timeless also appeared to play a role in the DNA damage checkpoint response, a process that arrests cell division and activates DNA repair mechanisms. Other experiments demonstrated that inhibiting production of Timeless protein seriously compromised checkpoint-regulated coordination of cell division, “indicating an intimate connection between the circadian cycle and DNA damage checkpoints.”

Although there is still much to be learned about the function and control of the Timeless protein, these results indicate that it does indeed act in the control of both the circadian clock and the cell cycle by interacting with circadian clock proteins and playing an important role in the DNA damage response. However, the authors point out that the circadian cycle operates normally in the absence of the cell cycle.

## Figures and Tables

**Figure:**